# Addressing the health workforce crisis: towards a common approach

**DOI:** 10.1186/1478-4491-4-21

**Published:** 2006-08-03

**Authors:** Mario R Dal Poz, Estelle E Quain, Mary O'Neil, Jim McCaffery, Gijs Elzinga, Tim Martineau

**Affiliations:** 1 Editor-in-Chief, *Human Resources for Health *online journal; 2 Department of Human Resources for Health, World Health Organization, Geneva, Switzerland; 3 Office of HIV/AIDS, United States Agency for International Development, Washington, DC, USA; 4 Management Sciences for Health, Boston, Massachusetts, USA; 5 Capacity Project, Intrahealth, Chapel Hill, North Carolina, USA; 6 National Institute for Public Health and the Environment, Bilthoven, The Netherlands; 7 Liverpool School of Tropical Medicine, Liverpool, UK

## Abstract

The challenges in the health workforce are well known and clearly documented. What is not so clearly understood is how to address these issues in a comprehensive and integrated manner that will lead to solutions. This editorial presents – and invites comments on – a technical framework intended to raise awareness among donors and multisector organizations outside ministries of health and to guide planning and strategy development at the country level.

## Background

The existence of a widespread health workforce crisis with the potential to reverse the health gains of the past decade and contribute to the collapse of the health system in some countries is generally acknowledged. The challenge is what to do about it. The *World health report 2006: Working together for health *asks: "How can a country begin to solve the myriad and complex problems of human resources for health that confront it? Is there a tool or a technical framework that will assist governments and national health workforce planners to develop and implement a comprehensive strategy in a systematic way?"

## Towards a common approach

The *World health report 2006 *presents an evolving technical framework to help countries resolve the problems underlying the crisis. The framework was developed by representatives of multilateral and bilateral agencies, donors, partner countries, nongovernmental organizations and the academic community who came together at an informal consultation sponsored by the World Health Organization and the United States Agency for International Development in Washington, DC, in December 2005. Their goal was to agree on the characteristics of a simple but comprehensive technical framework that would enable countries to develop a concrete national health workforce strategy that could be supported and implemented in a planned and systematic manner.

**Figure 1:**
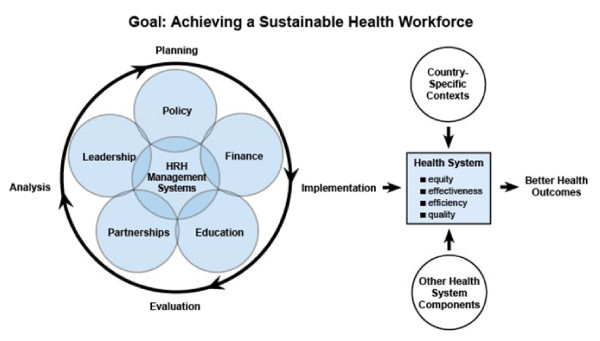


The resulting health workforce framework (Figure 1) presents six components of planning and managing the health workforce so that appropriately trained staff are available in the right places at the right time: health workforce management, policy, finance, education, partnerships and leadership. Health workforce management systems are at the center of the framework because of their importance in integrating all the other components.

Subsequent to the December 2005, a steering committee of participants in the consultative meeting and other health workforce experts have defined the six components and their subcomponents (Table 1).

**Table 1:** The six components of the health workforce framework, definitions thereof and the key elements in each

**Component**	**Definition**	**Subcomponents**
Policy	Rules, regulations & legislation for conditions of employment, work standards and development of the health workforce	• Professional standards, licensing, accreditation • Authorized scopes of practice for health workforce categories • Political, social & financial decisions that affect HRH • Employment law and rules for civil service
Health workforce management	Integrated use of data, policy and practice to plan for necessary staff, recruit, hire, deploy and develop health workers	• Personnel systems: workforce planning, recruitment, hiring, deployment. • Work environment & conditions: employee relations, workplace safety, job satisfaction, career development. • HRH information system • Performance management: performance appraisal, supervision, productivity. • Staff retention: financial & non-financial incentives.
Finance	Obtaining, allocating and dispersing adequate funding for human resources	• Salary and allowances • Budget for HRH • National HRH accounts with HRH information • Mobilizing financial resources (e.g, government, Global Fund, PEPFAR, donors)
Education	Production and continuous development of an appropriately skilled workforce	• Pre-service education tied to health needs • In-service training (e.g. distance and blended, continuing education) • Capacity of training institutions • Training of community health workers and non-formal care providers
Partnerships	Formal and informal linkages aligning key stakeholders, e.g. service providers, sectors, donors, to maximize use of human resources	• Community mobilization: supporting care and treatment, governance of health services. • Public – private sector agreements • Mechanisms and processes for multi-stakeholder cooperation (interministerial committees, health worker advisory boards, donor coordination groups)
Leadership	Capacity to provide direction, to align people, to mobilize resources and to reach goals	• Identify, select & support health workforce champions and advocates • Leadership development for health workforce managers at all levels • Capacity for multisector & sector-wide collaboration Modernizing & strengthening associations

## Benefits of the technical framework

The benefits of a comprehensive technical framework to the development and implementation of a sustainable health workforce include:

• identification of the key dimensions of technical competence needed to develop and implement a strategy for human resources;

• informing the growing number of groups interested in this area about the complexities of the health workforce and preventing the spread of simplistic and limited views on what is involved;

• providing a common reference point for all health workforce stakeholders and preventing policy-makers, implementers, donors, academics and others from "re-inventing the wheel".

Many actions can be taken to address each of the six components of the health workforce framework. Tools and guidelines will facilitate implementation of the interventions related to each of the six components. However, a fragmented approach to health workforce issues may be counter-productive and fail to result in sustainable change. While one intervention may concentrate on one or two of the components initially, it is crucial that a comprehensive plan be developed to integrate challenges in all six components. For example, it is essential to address the policy and financial implications of the crisis, but success in these components will not improve health services if the health workforce managers in the health facilities cannot turn these policy changes into practices that create positive workplace conditions. Likewise, even a well-managed health facility will not be able to cope with the overwhelming demands related to HIV/AIDS if partnerships are not formed with the community. Finally, leadership at all levels is critical for each component, to develop human capacity that can be sustained.

## Guiding principles

Table 2 presents a set of guiding principles developed to assist health workforce planners and implementers in applying the framework so as to serve the needs of a particular country best.

**Table 2:** Guiding principles for use of the health workforce framework

**Principle**	**Process-related**	**Principle**	**Content-related**
Country-led	Initiatives to improve the health workforce are carred forward by the country, rather than external partners	Results-focused	Health workforce strategies are aimed at achieving measurable improvements
Government-supported	Commitment by the government to support actions that contribute to a sustainable health workforce	System-linked	Health workforce strategies are harmonized with relevant components of the health system (e.g. monitoring & evaluation, supply chain, finance)
Multisectoral	Engagement by all sectors relevant to building the health workforce (e.g. finance, education, public & private providers, etc.)	Knowledge-based	Decisions are based on best available documented health workforce experience
Multistakeholder	Inclusion of interest groups relevant to particular actions (e.g. NGOs, patient groups, professional associations, donor coordinating committees, etc.)	Learning-oriented	Uses monitoring & evaluation to identify lessons learnt and best practices to share within and outside countries
Donor alignment	Donor support coordinated and aligned with country health workforce plans	Innovation-prone	Openness to exploring new solutions to overcome chronic health workforce issues
Gender-sensitive	Gender differences accounted for in analysis and development of health workforce strategies		

## Next steps

As noted, the steering committee is now incorporating tools and guidelines – the value of which has been or will be assessed – in the health workforce framework to enhance its operational value for planning and implementing strategies and policies to strengthen the health workforce.

The final product will be a web-based version of the framework by the end of 2006, with versions on CD-ROM and in print to promote in-country use and ownership. As the health workforce field is continuously evolving and changing, this framework will be continuously adapted and upgraded. In the same spirit, all health workforce practitioners, experts and researchers are invited to respond, in an open and participatory way, to this editorial.

## Competing interests

The author(s) declare that they have no competing interests.

## References

[B1] Joint Learning Initiative (2004). Human Resources for Health: Overcoming the Crisis.

